# Duration of Static and Dynamic Periods of the Upper Arm During Daily Life of Manual Wheelchair Users and Matched Able-Bodied Participants: A Preliminary Report

**DOI:** 10.3389/fspor.2021.603020

**Published:** 2021-03-26

**Authors:** Brianna M. Goodwin, Omid Jahanian, Stephen M. Cain, Meegan G. Van Straaten, Emma Fortune, Melissa M. Morrow

**Affiliations:** ^1^Division of Health Care Delivery Research, Mayo Clinic, Rochester, MN, United States; ^2^Robert D. and Patricia E. Kern Center for the Science of Health Care Delivery, Mayo Clinic, Rochester, MN, United States; ^3^Department of Mechanical Engineering, University of Michigan, Ann Arbor, MI, United States; ^4^Assistive and Restorative Technology Laboratory, Rehabilitation Medicine Research Center, Department of Physical Medicine and Rehabilitation, Mayo Clinic, Rochester, MN, United States

**Keywords:** inertial measurement units, remote data capture, arm intensities, spinal cord injury, humeral elevation, shoulder

## Abstract

**Background:** Manual wheelchair (MWC) users with spinal cord injuries (SCI) are at a significantly higher risk of experiencing rotator cuff pathology than able-bodied individuals. A deeper understanding of where the arm is used dynamically within the humeral workspace during daily life may help explain why MWC users have higher shoulder pathology rates than able-bodied individuals. The purpose of this study was to report the daily percentage and consecutive durations MWC users and matched able-bodied individuals (controls) spent static and dynamic across the humeral elevation workspace.

**Methods:** MWC users with SCI and controls wore three inertial measurement units on their bilateral arms and torso for 1 or 2 days. The percentages of time and average consecutive duration individuals were static or dynamic while in five humeral elevation ranges (0–30°, 30–60°, 60–90°, 90–120°, and >120°) were calculated and compared between cohorts.

**Results:** Forty-four MWC users (10 females, age: 42.8 ± 12.0, time since injury: 12.3 ± 11.5) and 44 age- and sex-matched controls were enrolled. The MWC cohort spent significantly more time dynamic in 60–90° (*p* = 0.039) and 90–120° (*p* = 0.029) and had longer consecutive dynamic periods in 30–60° (*p* = 0.001), 60–90° (*p* = 0.027), and 90–120° (*p* = 0.043) on the dominant arm. The controls spent significantly more time dynamic in 0–30° of humeral elevation (*p* < 0.001) on both arms. Although the average consecutive static durations were comparable between cohorts across all humeral elevation ranges, the MWC cohort spent a significantly higher percentage of their day static in 30–60° of humeral elevation than controls (dominant: *p* = 0.001, non-dominant: *p* = 0.01). The MWC cohort had a moderate association of increased age with decreased time dynamic in 30–60° for both arms.

**Discussion:** Remote data capture of arm use during daily life can aid in understanding how arm function relates to shoulder pathology that follows SCI and subsequent MWC use. MWC users spent more time dynamic in higher elevations than controls, and with age, dynamic arm use decreased in the 30–60° humeral elevation range. These results may exemplify effects of performing activities from a seated position and of age on mobility.

## Introduction

It is estimated that over a quarter of a million people have a spinal cord injury (SCI) in the United States, with over 17,000 new cases occurring each year (NSCISC, 2020). Many individuals with SCI use a manual wheelchair (MWC) as their main mode of mobility. Consequently, the arms are required for both mobility and activities of daily living (ADLs). Among this population, the shoulder is the most common site of musculoskeletal pain and pathology, with the reported prevalence ranging between 40 and 70% (Curtis et al., 1999; Dalyan et al., 1999; Dyson-Hudson and Kirshblum, 2004; Alm et al., 2008; Divanoglou et al., 2018). Although the risk of degenerative shoulder pathology increases with age for able-bodied individuals (Leong et al., 2019), pathology progression is accelerated for MWC users (Akbar et al., 2010). In a sample of 100 chronic MWC users and 100 age-matched able-bodied adults (henceforth, referred to as controls), MWC users were reported to have a four times higher prevalence of having a rotator cuff tear compared to controls (Akbar et al., 2010). Understanding how MWC users use their arms during daily life and how this differs from controls may aid in understanding why MWC users are at a higher risk for increased shoulder pain and pathology. Remote data collections allow for a unique opportunity to capture a holistic view of arm use in the free-living environment and ultimately uncover patterns of movement which contribute to increased shoulder pain and pathology.

Multiple groups have used remote data capture techniques to understand the way MWC users move and how this movement is associated with shoulder pain. MWC propulsion characteristics (metrics: median propulsion bout and daily durations and distances traveled) were measured with a wheel mounted accelerometer during daily life to conclude that short, slow bouts dominate daily wheelchair usage (Sonenblum et al., 2012). Daily task (wheelchair propulsion, caretaker pushing, wheelchair basketball, household chores, etc.) classification algorithms have been developed based on input data from accelerometers and inertial measurement units (IMU) affixed to the body and wheelchair, with the long-term goal to give wheelchair users real-time feedback (Hiremath et al., 2015; Fortune et al., 2019). Remote data capture using a sensor on the wheel (metrics: daily distance traveled and average speed) and phone interviews to recall daily events (metrics: number of transfers and hours of sport participation) were investigated in parallel with pain occurrence in one study (Mulroy et al., 2015). Mulroy et al. (2015) suggest that propulsion speed, daily propulsion distance, number of transfers, and weekly hours of sport participation were not associated with increased shoulder pain. Rather, MWC users who were less active were more likely to develop shoulder pain. The remote data capture methodologies presented here fill a gap in the literature by measuring shoulder kinematics, specifically static and dynamic humeral elevations, during free-living of manual wheelchair users.

Our group's investigation into the link of arm use and shoulder pain and pathology has focused on differentiating arm use patterns during daily life between MWC users and matched controls with the goal of explaining the striking difference in shoulder pain and pathology between groups. We have previously used IMUs in the free-living environment to capture the humeral elevation workspace during typical days for these two cohorts (Goodwin et al., 2020), and we have investigated the use of a threshold to decipher static from dynamic arm use during daily life (Goodwin et al., 2021). The motivation for this line of investigation is evidence-based knowledge that factors that affect overall health and damage to the rotator cuff tendons are multifactorial, ultimately resulting from different types of motion and loading patterns (Lewis, 2010; McCreesh and Lewis, 2013). Maintaining rotator cuff tendon health relies on a sensitive balance of exposure to motion and loading patterns that do not exceed the mechanical and biological capacity of the tendon. By investigating arm use using metrics such as static and dynamic periods across the humeral elevation workspace, we can estimate the exposure of the shoulder to factors that can results in tendon pathology over time by going beyond tendon capacity. For example, static time provides insight into periods of rest when static time occurs at low humeral elevations and isometric loading of the arm when static time occurs at higher elevations. Dynamic arm use provides insight into potential repetitive motion and use of the arm that may or may not include additional loading applied at the hand. Further, understanding the consecutive duration of the static and dynamic periods during daily life highlights and can explain how the arm is being used to perform activities of daily living.

The purpose of this study was to report the percentage of daily time that the upper arms of MWC users and matched controls were static or dynamic across the humeral elevation workspace (0–30°, 30–60°, 60–90°, 90–120°, and >120°). We also wanted to investigate differences in the average duration of consecutive seconds of static and dynamic time between cohorts. This analysis adds context when interpreting differences in overall daily percentages between cohorts. Further, we aimed to investigate the effects of age and shoulder pain on the percentage of time the upper arm was static or dynamic in each humeral elevation range for both cohorts. We hypothesized that due to the different ADLs required throughout a day for MWC users and controls, the cohorts would have an overall different distribution of static and dynamic periods across the humeral elevation workspace. We also hypothesized that age (both cohorts) and pain (continuous variable for the MWC cohort and binary variable for both cohorts) would be negatively associated with time dynamic across the humeral elevation ranges.

## Methods

### Participant Recruitment

This study was approved by the Mayo Clinic Institutional review board. This study was part of a larger study (Natural History of Shoulder Pathology in Wheelchair Users) that follows the longitudinal development of shoulder pathology on MRI and arm use during daily life in adults with SCI who are MWC users and matched able-bodied participants. Prior to study accrual, a licensed physical therapist (co-author MVS) performed a screening physical exam to confirm inclusion and exclusion criteria on both cohorts. Inclusion criteria for individuals in the MWC cohort included: being between the ages of 18 and 70; use of a MWC as their main mode of mobility; functional upper extremity range of motion, defined as active humeral thoracic flexion and abduction of at least 150° and the ability of the participant to touch the opposite shoulder, the back of his/her neck and his/her low back; willingness to participate in study activities, and the ability to return to Mayo Clinic to receive shoulder MRI annually for 3 years, and once per year receive physical exam. Exclusion criteria for the MWC cohort included previous diagnosis of bilateral supraspinatus tendon tears prior to SCI or conditions/factors which may have hindered protocol adherence. MWC cohort participants were recruited by querying medical records and referrals by care providers of local clinics. The inclusion criteria for the sex- and age-matched (±3 years) able-bodied controls included the same age, functional upper extremity, and visit attendance criteria as the MWC cohort. Additionally, the matched controls had to have the ability to walk independently with no reliance on an orthotic, prosthetic, or gait aid. Exclusion criteria for the matched controls included: any documented musculoskeletal or neurological disorders that would be expected to impact shoulder health or change ability to walk independently; previous diagnosis of unilateral or bilateral supraspinatus tears prior to enrollment; or conditions/factors which may have hindered protocol adherence. Able-bodied matches to the MWC cohort were recruited through email distribution lists and classified ads.

### Surveys and Questionnaires

Prior to enrollment, written informed consent was obtained during an in-person meeting. All participants were asked if they had pain in either of their shoulders (binary, presence of pain). Additionally, pain was measured on a continuous scale for the MWC cohort using the Performance Corrected Wheelchair User Shoulder Pain Index (PC-WUSPI) for both the right and left shoulders. The performance correction was used because not all participants completed all tasks. To calculate the PC-WUSPI the WUSPI scores were divided by the number of questions the participant completed and multiplied by 15. The WUSPI was originally designed to be completed once per patient/participant while considering overall shoulder pain; however, in this study participants completed the survey twice, once for each shoulder. To complete the WUSPI, participants rated their shoulder pain when completing 15 tasks on a visual analog scale between “no pain” and “worst pain ever experienced,” or they indicated if they did not perform the activity (Curtis et al., 1995a). Possible scores ranged from 0 (no pain) to 150 (worst pain ever experienced in all categories). The WUSPI is valid and reliable for this population (Curtis et al., 1995b).

### Remote Data Collections

**Figure 1:**
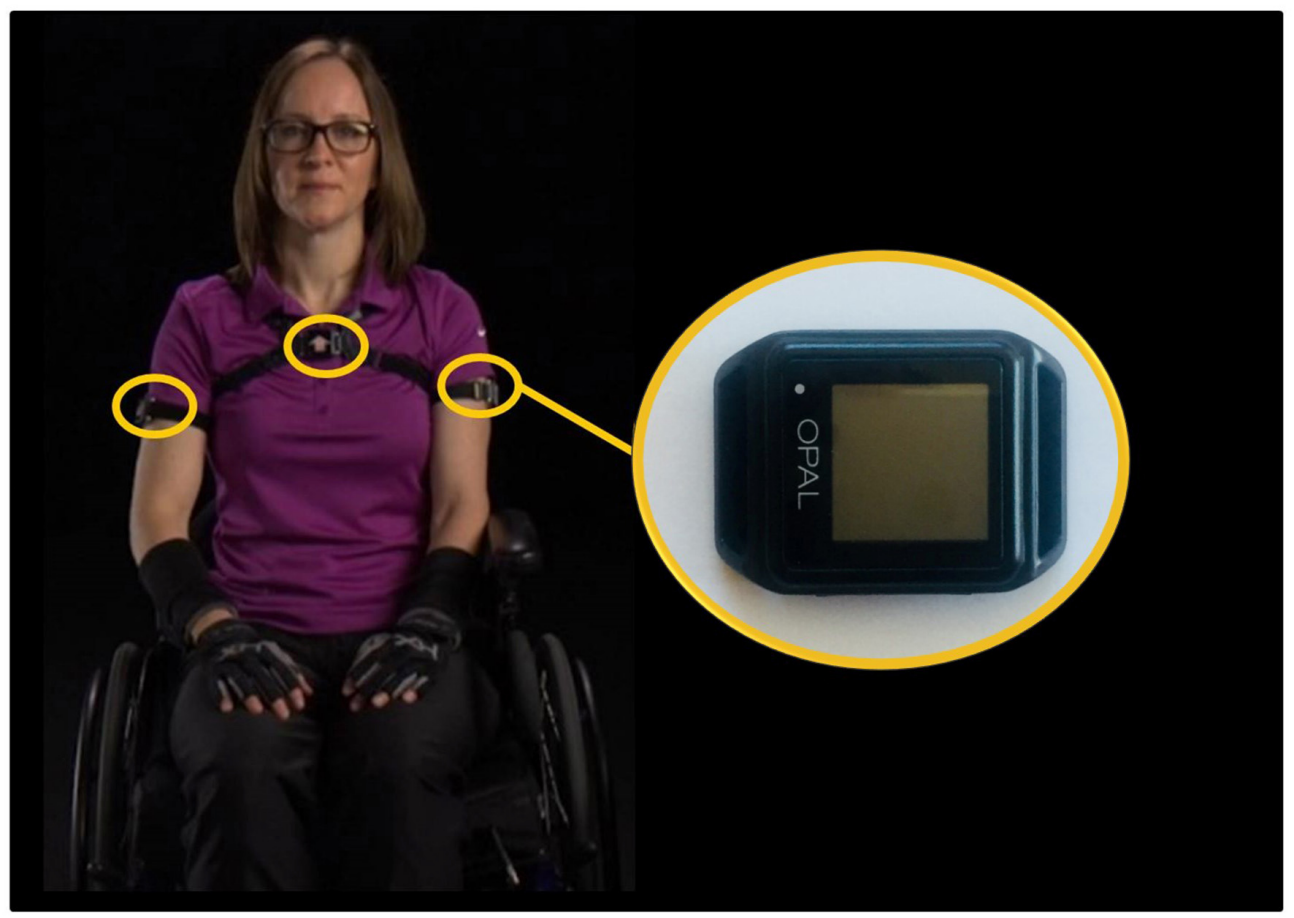
The inertial measurement units were worn by participants on their bilateral upper arms and torso. Note the individual pictured here is a study team member.

Participants were provided three wireless IMUs (Opal or Emerald, APDM Inc., Portland, Oregon). Each IMU collected 3-axis accelerometer (±16 g and ±200 g), 3-axis gyroscope (±2000°/s), and 3-axis magnetometer (±8 Gauss) data at 128 Hz. The IMUs remained synchronized via a manufacturer provided proprietary algorithm (APDM, 2020). Participants were instructed to wear the IMUs on each of their lateral upper arms and torso; elastic Velcro straps were used to secure the IMUs to the body (Figure 1). To maximize the consistency of IMU placement each IMU was labeled with the proper wear location (left arm, right arm, or torso) and an arrow indicating the proper mounting orientation. Participants were instructed to wear the upper arm sensors on the lateral aspect of their arm above the muscle belly of the biceps, and the chest sensor was to be worn on the chest in the middle of the sternum. Further, participants were instructed to tighten all straps to prevent movement of the sensor on the body while still maintaining comfort. Participants were asked to wear the sensors for two typical days for at least 8 h, excluding bathing and swimming, and take them off before going to bed. Participants were also asked to charge the sensors overnight (between data collections) and perform a functional calibration at the beginning of each day and after re-donning the sensors if they were taken off in the middle of the day (Goodwin et al., 2020). Both cohorts were instructed to perform their regular daily routines; the control cohort did not use MWCs. To increase protocol adherence all participants were provided in-person, written, and video instructions. Participants were also provided with a pre-paid envelope or met study staff in person to return the sensors after completion of the data collection.

### Data Processing

Raw acceleration and estimates of orientation relative to an inertial frame data were downloaded through Motion Studio (APDM, Inc., Portland, Oregon). Orientation estimates, using the acceleration and gyroscope data, were calculated in Motion Studio without magnetometer data due to the likely non-uniform and unknown magnetic fields present throughout the field data collections. While the orientation algorithm used by APDM is proprietary, sensor fusion methods (e.g., Kalman filters) used to estimate IMU orientation from raw sensor data are well-understood and well-documented in the literature (Savage, 1998; Sabatini, 2006; El-Gohary and McNames, 2012).

Custom MATLAB (Mathworks, Natick, Massachusetts) code was written to calculate the humeral elevation and static vs. dynamic status of each second of the day. This process is described in detail elsewhere (Goodwin et al., 2020, 2021). In short, the signal magnitude area (SMA) of the raw acceleration data was calculated for each second and used to determine if each arm was static or dynamic. The acceleration signal was filtered with a centered median filter to reduce noise spikes (window size of three frames) (Karantonis et al., 2006; Lugade et al., 2014). The gravitational component of the signal was then calculated by using a third-order zero phase lag elliptical low pass filter with 0.25 Hz cut-off frequency, 0.01 dB passband ripple and −100 dB stopband ripple. The gravitational component of the acceleration signal was subtracted from the original signal to leave the acceleration due to body motion, and then the SMA was calculated for each second of data (Lugade et al., 2014). A threshold between static and dynamic arm movement (SMA ≤ 0.67 g) was calculated as part of a previous study and applied to the data in the present study (Goodwin et al., 2021).

The humeral elevation angles were calculated using the functional calibration to align the inertial reference frame with the anatomical axes. Humeral elevation and thorax deviation angles were defined as the angle between the long axis of the body segment (defined from the function calibration) and vertical; these angles are only dependent on the estimated direction of gravity relative to the body segment and, therefore, are drift-free metrics for quantifying body segment motions (Goodwin et al., 2020). The humeral elevation angles have previously been validated in unpublished data where five individuals with SCI performed 10 reaching tasks. The absolute error and percent of error when compared to the gold standard (electromagnetic system) were −0.06 ± 1.12° and −1.44 ± 1.28%, respectively, for the range of motion. The absolute error and percent of error for the maximum elevation achieved during each reach were 2.59 ± 2.47° and 2.04 ± 2.47%, respectively. The average humeral elevation angle was calculated for each second; possible angles range between 0 and 180°, with 0° indicating the arm was down and aligned with gravity and 180° indicating the arm was raised overhead and aligned with gravity. Each second of the data was classified as static or dynamic in one of the humeral elevation ranges (0–30°, 30–60°, 60–90°, 90–120°, and >120°). The humeral elevation angle was calculated with respect to the neutral humerus position and not with respect to the thorax; therefore, we eliminated data when the average torso angle was over 30° (Goodwin et al., 2020). By eliminating data where the trunk was at high deviation angles from upright, periods where the humeral elevation angle was likely much different than the humerothoracic angle were not included in the analysis.

### Outcome Measure Definitions

Two primary outcome measures were calculated based on the second-by-second data: the percentage (%) and total duration of the full day that each arm was static or dynamic in each of the five humeral elevation ranges, and the average consecutive duration of static and dynamic periods in each humeral elevation range. In the first analysis, the sum of all static and dynamic percentages equals 100% of the full data collection. The duration that the arms were static or dynamic in each humeral elevation range was calculated by multiplying the percentage of daily time spent static or dynamic in each elevation range by the average length of all files (MWC and cohort cohorts both included). The average consecutive periods were calculated by determining the duration of all consecutive static or dynamic periods in each humeral elevation range. The average duration each participant spent static or dynamic in each humeral elevation range was calculated, followed by the calculation of group statistics.

### Requirements for Inclusion of Data in Analysis

Data files were excluded if <8 h of useable data were collected for each day or participants did not complete the daily functional calibration; 8 h was chosen as a threshold as a balance between sensor battery life and inclusion of data. Although instructed to collect 2 days of data, in some cases only 1 day of data was collected or 2 days were collected, but 1 day was not useable. If only 1 day of usable data existed, metrics were calculated from the single day. If participants provided 2 days of usable data, the data were concatenated and the percentages were calculated using all available data.

### Statistical Analysis

Statistical analyses were performed in SPSS 25 (IBM Corp., Armonk, NY). Non-parametric tests were used due to the non-homogeneity in participant demographics (age, sex, level of SCI) and visually inspected non-normal distributions. A Kruskal-Wallis test was used to test for the effect of cohort (MWC vs. control, primary predictor) on the percentage of daily time static or dynamic and the average consecutive time in each humeral elevation range for both the dominant and non-dominant arms. Kruskal-Wallis tests were also used to test for differences between the dominant and non-dominant arm. When significant differences were observed, *post-hoc* analyses were completed using separate Wilcoxon Signed Ranks test. Differences between participants with and without shoulder pain (binary variable) were compared using separate Mann-Whitney U tests for the main outcomes (daily percentage spent static and dynamic). For the primary aim, the effect size was calculated as the ratio of the z-value to the square root of the number of participants. No statistics were used to compare the total duration of time (in minutes) static or dynamic in each humeral elevation range, as this was only used to give context to the percentage of daily time in each range and calculated from each percentage. Additionally, Spearman's correlation was used to investigate the association between age (both cohorts) and pain (measured by the PC-WUSPI, MWC cohort only) with the percentage of time spent static or dynamic in each humeral elevation range and the average consecutive time in the range. Correlation coefficients between 0.1 and 0.29 were considered small association, between 0.3 and 0.49 were considered moderate association, and equal to or above 0.5 were considered large association (Cohen, 2013). *P*-values < 0.05 were considered statistically significant in all tests.

## Results

Forty-four participants with SCI who use a MWC and 44 age- (±3 years) and sex-matched controls were enrolled (Table 1). Participants with SCI were independent in their mobility and self-care. There were missing data for one participant in the MWC cohort and two participants in the control cohort for the presence/absence of pain. Thirty-three participants in the MWC cohort collected 2 days of useable data and 11 participants in this cohort only collected 1 day of useable data. The second day of data were excluded because it did not meet the 8 h minimum criteria (six participants), a sensor malfunctioned (two participants), and only 1 day of data was collected (three participants). Forty participants in the control cohort collected 2 days of useable data. The second day of control participant data were excluded in four cases because only 1 day of data was collected (one participant) and the collected data did not meet the 8 h minimum (three participants). Supplementary Material A shows the participant demographics stratified by the number of days of data collected. The MWC and control cohorts averaged 9.1 ± 2.0 and 8.3 ± 1.3 h of data collected per day, respectively. Overall an average of 8.7 h was used to estimate the time spent static or dynamic in each humeral elevation range.

**Table 1:** Participant demographics.

	**MWC**	**Control**	***P*-value**
Age (years)			
Mean (SD)	42.8 (12.0)	42.8 (11.8)	-
Median (IQR)	39.3 (34.3–54.9)	39.9 (33.5–54.5)	-
Sex	10 females/34 males	10 females/34 males	-
Self-reported weight (kg)			
Mean (SD)	77.9 (14.1)	82.5 (16.2)	0.121^a^
Median (IQR)	77.1 (65.8–88.6)	81.6 (72.1–88.9)	
Self-reported height (cm)			
Mean (SD)	176.9 (8.3)	176.9 (10.1)	0.743^a^
Median (IQR)	177.8 (172.7–180.6)	177.8 (170.2–182.9)	
Body mass index (kg/m^2^)			
Mean (SD)	24.9 (4.3)	26.3 (4.4)	0.116^a^
Median (IQR)	24.6 (22.3–27.0)	25.2 (23.6–28.9)	
Dominant arm	36 right/8 left	39 right/5 left	0.179^b^
Injury Level			
Cervical (C6–C7)	8	-	-
High/mid thoracic (T1–T8)	18		
Low thoracic/lumbar (T9–L1)	18		
Time since injury (years)			
Mean (SD)	12.3 (11.5)	-	-
Median (IQR)	6.8 (3.4–20.8)		
PC-WUSPI (dominant arm)			
Mean (SD)	16.1 (26.3)	-	-
Median (IQR)	3.7 (0.1–16.7)		
PC-WUSPI (non-dominant arm)			
Mean (SD)	16.2 (22.9)	-	-
Median (IQR)	7.7 (0.0–19.4)		
Number of participants who reported pain	31 (70%)	8 (18%)	<0.001^b^
Dominant	4 (9%)	1 (2%)	
Non-dominant	6 (14%)	2 (5%)	
Bilateral	21 (47%)	5 (11%)	

SD, standard deviation; IQR, inter-quartile range.

a Wilcoxon Signed Ranks Test.

b *Chi Squared Test*.

### Percentage and Duration of Static and Dynamic Periods in Each Humeral Elevation Range

**Figure 2:**
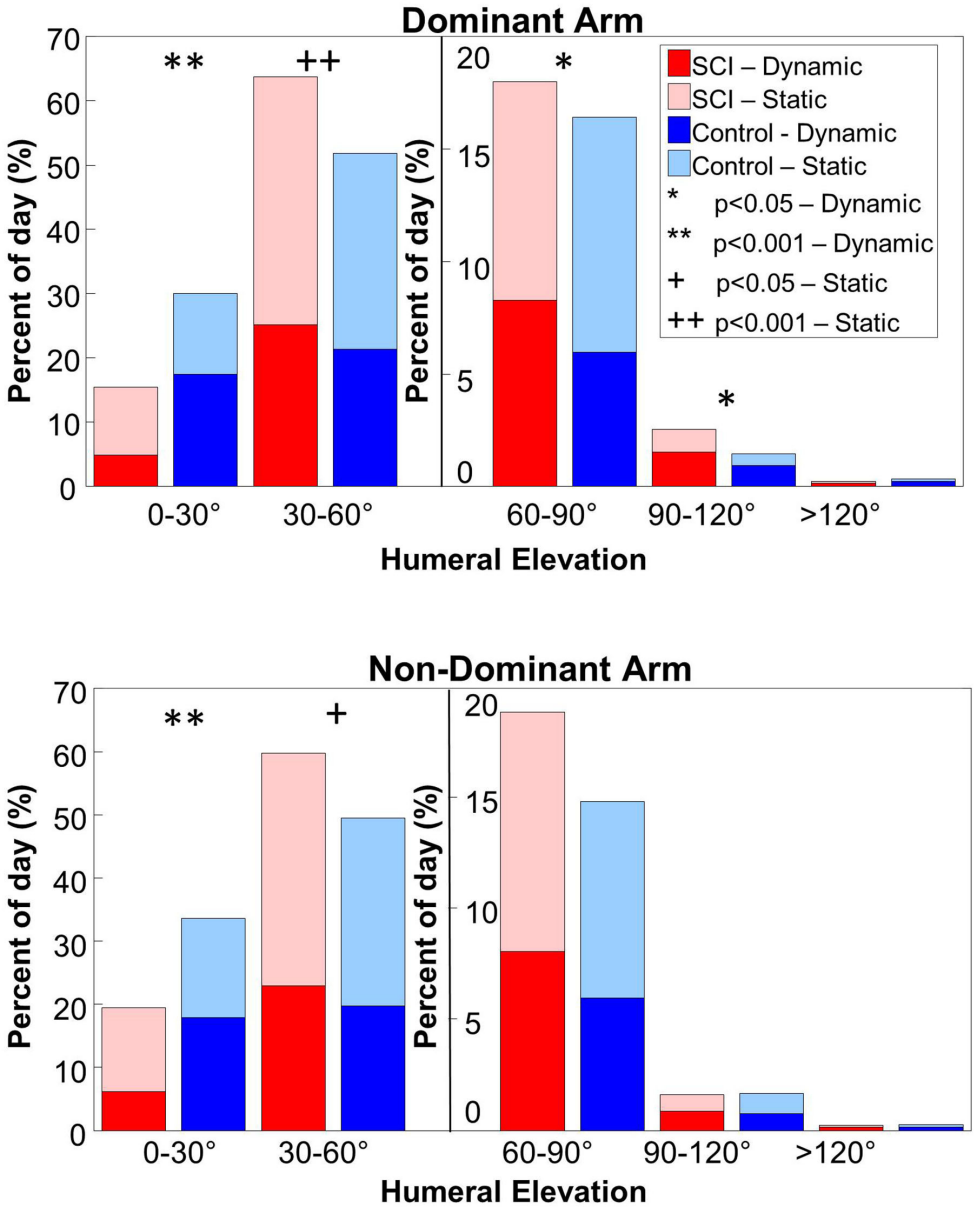
The percent of the day the MWC (red colors) and control (blue colors) cohorts spent static and dynamic in each humeral elevation range for the dominant (top pane) and non-dominant (bottom pane) arms. The lighter colors indicate static time and the darker colors indicate dynamic time. A secondary y-axis is used for humeral elevations over 60° to more clearly show the differences and similarities between cohorts. Analyses were conducted between the MWC and control cohorts for the static periods (+ indicates *p* < 0.05 and ++ indicates *p* < 0.001) and dynamic periods (* indicates *p* < 0.05 and ** indicates *p* < 0.001).

There was a significant main effect of cohort on the percentage of daily time static or dynamic in each humeral elevation range (*p* < 0.001). *Post-hoc* tests revealed the MWC cohort spent significantly more time dynamic on the dominant side in 60–90° and 90–120° of humeral elevation than the matched controls (Figure 2; Table 2). On average, the arms' of MWC users were dynamic for ~30 more minutes per day between 30 and 90° of humeral elevation than the matched controls. However, the control cohort spent a significantly higher percentage of the day (~12% of the day or 60 min per day) dynamic in 0–30° of humeral elevation on both dominant and non-dominant sides. On average, the MWC cohort spent over 7.5% more of their daily time, ~40 min, static in 30–60° of humeral elevation. There were no differences between dominant and non-dominant arms for either cohort. Box plots to show the data variability are available in the Supplementary Material section B.

**Table 2:** The percentage of the day (and minutes) the MWC and control cohorts spent static and dynamic in each humeral elevation range.

	**MWC Mean (%) ± standard deviation (time)**	**Control** **Mean (%) ± standard deviation (time)**	**Difference (MWC–Control) Mean (%) (time)**	***p*-value^a^**	**z score**	**Effect size**
**Dominant arm–Static**						
0–30°	10.5 ± 9.4 (55.0 min)	12.5 ± 7.5 (65.3 min)	−2.0 (−10.3 min)	0.172	−1.365	−0.146
30–60°	38.6 ± 13.4 (201.4 min)	30.4 ± 10.2 (158.9 min)	8.2 (42.5 min)	0.001*	−3.338	−0.356
60–90°	9.7 ± 9.5 (50.7 min)	10.4 ± 7.7 (54.5 min)	−0.3 (−3.8 min)	0.427	−0.794	−0.085
90–120°	1.0 ± 2.2 (5.2 min)	0.5 ± 0.6 (2.6 min)	0.5 (2.6 min)	0.299	−1.039	−0.111
>120°	0.1 ± 0.2 (0.4 min)	0.1 ± 0.2 (0.6 min)	0.0 (−0.2 min)	0.971	−0.036	−0.004
**Dominant arm–Dynamic**						
0–30°	4.9 ± 5.2 (25.8 min)	17.5 ± 8.9 (91.2 min)	−12.6 (−65.4 min)	<0.001*	−5.532	−0.590
30–60°	25.2 ± 9.4 (131.3 min)	21.4 ± 7.6 (111.6 min)	3.8 (19.7 min)	0.070	−1.809	−0.193
60–90°	8.3 ± 4.3 (43.2 min)	6.0 ± 3.3 (31.2 min)	2.3 (12.0 min)	0.039*	−2.066	−0.220
90–120°	1.5 ± 2.5 (8.0 min)	0.9 ± 1.3 (4.9 min)	0.6 (3.1 min)	0.029*	−2.182	−0.233
>120°	0.1 ± 0.2 (0.7 min)	0.2 ± 0.5 (1.1 min)	−0.1 (−0.4 min)	0.575	−0.56	−0.060
**Non-dominant arm–Static**						
0–30°	13.3 ± 11.8 (69.4 min)	15.6 ± 8.9 (81.7 min)	−2.3 (−12.3 min)	0.253	−1.144	−0.122
30–60°	36.8 ± 15.1 (191.9 min)	29.8 ± 10.8 (155.5 min)	7.0 (36.4 min)	0.010*	−2.579	−0.275
60–90°	10.8 ± 13.2 (56.6 min)	8.9 ± 5.4 (46.4 min)	1.1 (10.2 min)	0.861	−0.175	−0.019
90–120°	0.8 ± 1.2 (3.9 min)	0.9 ± 2.7 (4.9 min)	−0.1 (−1.0 min)	0.649	−0.455	−0.049
>120°	0.1 ± 0.1 (0.4 min)	0.1 ± 0.2 (0.6 min)	0.0 (−0.2 min)	0.512	−0.656	−0.070
**Non-dominant arm–Dynamic**						
0–30°	6.2 ± 5.6 (32.2 min)	18.0 ± 9.8 (93.7 min)	−11.8 (−61.5 min)	<0.001*	−5.322	−0.567
30–60°	23.0 ± 10.3 (119.9 min)	19.7 ± 8.8 (103.1 min)	3.3 (16.8 min)	0.176	−1.354	−0.144
60–90°	8.1 ± 4.8 (42.3 min)	6.0 ± 3.2 (31.3 min)	2.1 (11.0 min)	0.069	−1.821	−0.194
90–120°	0.9 ± 0.7 (4.6 min)	0.8 ± 0.5 (4.0 min)	0.1 (0.6 min)	0.407	−0.829	−0.088
>120°	0.2 ± 0.3 (0.8 min)	0.2 ± 0.2 (0.9 min)	0.0 (−0.1 min)	0.165	−1.389	−0.148

Bolded text and an asterisk next to the p-value indicate statistical significance at p < 0.05.

a Wilcoxon Signed Ranks Test.

* *Statically significant (p < 0.05)*.

**Figure 3:**
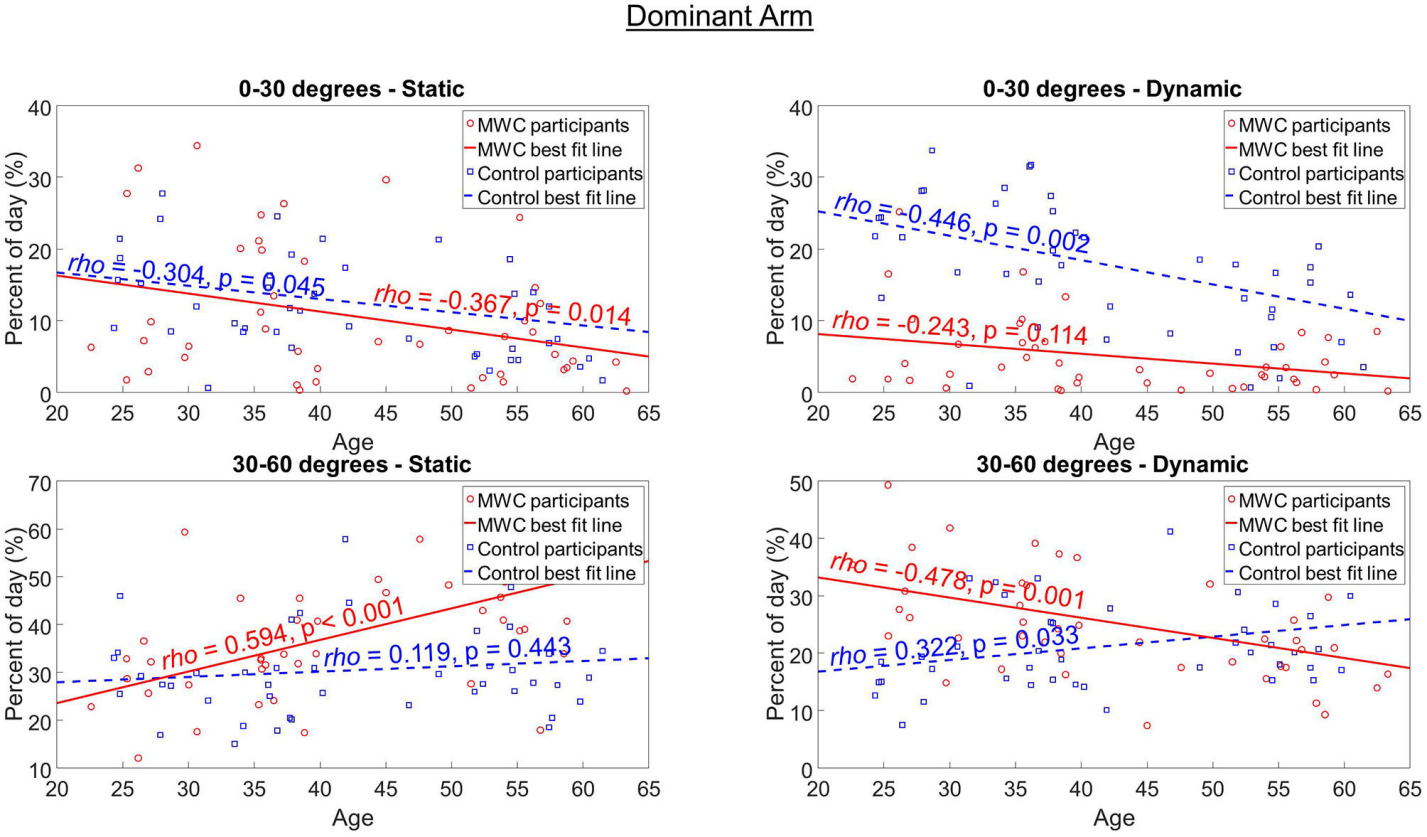
The association between age and the percentage of the day static and dynamic in 0–30° and 30–60° of humeral elevation on the dominant arm for MWC (red circles and red solid line) and control (blue squares and blue dashed line) cohorts. The Spearman's Correlation *rho* (ρ) denotes the strength of the association and the *p*-value shows the significance. Figures of the additional humeral elevation ranges for the dominant and non-dominant arms can be found in the Supplementary Material sections C and D, respectively.

Age was significantly associated with the percentage of the day spent static or dynamic in multiple humeral elevation ranges for both cohorts. For the MWC cohort, there were associations of decreased dynamic time as age increases in multiple humeral elevation bins [Figure 3; Table 3, Supplementary Material C (dominant arm) and D (non-dominant arm)]. The MWC cohort had large and moderate associations of increased age with an increased percentage of their day static in 30–60° of humeral elevation on the dominant and non-dominant sides, respectively (Figure 3; Table 3). The MWC cohort also had moderate association of increased age with decreased time dynamic in 30–60° and static in 0–30°. Finally, there were similar moderate levels of correlation between increased age and a decreased percentage of time dynamic in 0–30° for both cohorts on the non-dominant side. For both cohorts, none of the outcome measures were significantly different between those with and without pain. There were also no significant associations of the percentage of time spent static or dynamic in any humeral elevation ranges with the PC-WUPSI scores.

**Table 3:** The correlation coefficient between age and the percentage of day spent static or dynamic in each humeral elevation range.

	**MWC**		**Control**	
	**Correlation Coefficient^a^**	***p*-value**	**Correlation Coefficient^a^**	***p*-value**
**Dominant arm–Static**				
0–30°	−0.304	0.045*	−0.367	0.014*
30–60°	0.594	<0.001*	0.119	0.443
60–90°	0.225	0.142	0.214	0.163
90–120°	−0.09	0.561	0.090	0.560
>120°	−0.176	0.252	−0.047	0.762
**Dominant arm–Dynamic**				
0–30°	−0.243	0.114	−0.446	0.002
30–60°	−0.478	0.001*	0.322	0.033*
60–90°	−0.202	0.188	0.167	0.278
90–120°	−0.274	0.072	−0.069	0.658
>120°	−0.239	0.118	−0.059	0.703
**Non-dominant arm–Static**				
0–30°	−0.145	0.347	−0.200	0.193
30–60°	0.483	0.001*	0.211	0.170
60–90°	0.166	0.282	0.137	0.374
90–120°	−0.127	0.410	−0.053	0.732
>120°	−0.333	0.027	0.137	0.373
**Non-dominant arm–Dynamic**				
0–30°	−0.300	0.048*	−0.344	0.022*
30–60°	−0.424	0.004*	0.224	0.144
60–90°	−0.232	0.129	0.151	0.327
90–120°	−0.253	0.098	0.022	0.889
>120°	−0.264	0.084	−0.031	0.840

Bolded text and an asterisk next to the p-value indicate statistical significance at p < 0.05.

a Spearmans' Correlation.

* *Statistically significant (p < 0.05)*.

### Average Consecutive Static and Dynamic Periods

**Figure 4:**
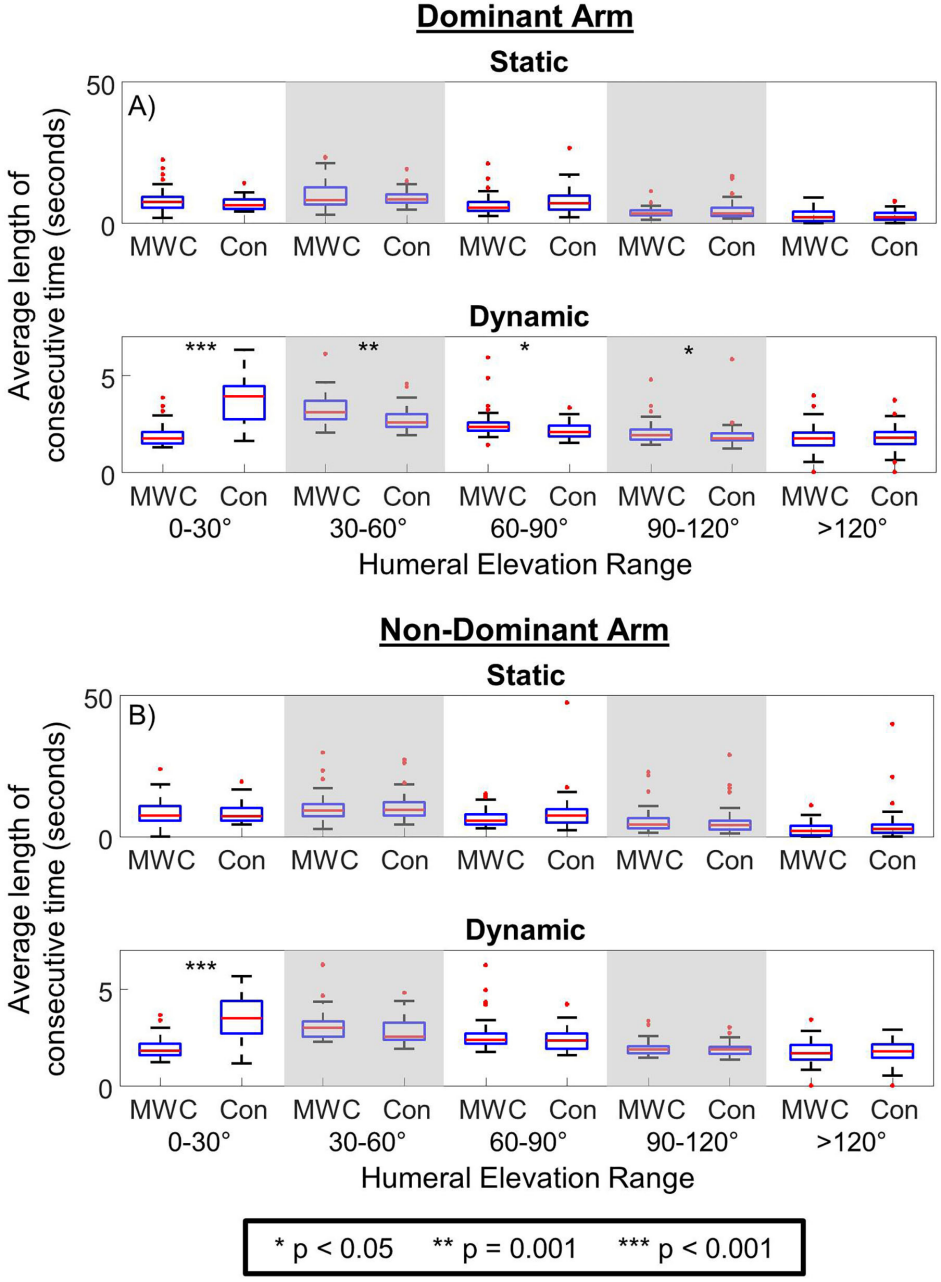
The average consecutive duration of static and dynamic periods in each humeral elevation range for MWC (MWC) and controls (Con) cohorts for the dominant arm **(A)** and non-dominant arm **(B)**. * indicates *p* < 0.05, ** indicates *p* = 0.001, and *** indicates *p* < 0.001.

There were differences in the average duration of consecutive seconds of dynamic periods between cohorts for all elevation ranges except >120° on the dominant side (Figure 4; Table 4). The control cohort had significantly longer average consecutive dynamic periods in 0–30° than MWC users on both the dominant and non-dominant sides. On the dominant side, the MWC users had longer consecutive durations of dynamic use than controls in 30–60°, 60–90°, and 90–120°. There were no differences between cohorts in consecutive static periods.

**Table 4:** The average consecutive duration of static and dynamic periods in each humeral elevation range.

	**MWC** **Mean consecutive seconds ± standard deviation**	**Control** **Mean consecutive seconds ± standard deviation**	***p*-value^a^**	**z-score**	**Effect size**
**Dominant–Static**					
0–30°	8.0 ± 4.4	6.8 ± 2.3	0.225	−1.214	−0.129
30–60°	9.8 ± 4.5	8.9 ± 3.0	0.484	−0.700	−0.075
60–90°	6.3 ± 3.5	7.8 ± 4.5	0.105	−1.622	−0.173
90–120°	3.6 ± 1.7	4.5 ± 3.3	0.278	−1.085	−0.116
>120°	2.5 ± 2.2	2.5 ± 1.9	0.957	−0.054	−0.006
**Dominant–Dynamic**					
0–30°	1.9 ± 0.6	3.7 ± 1.1	<0.001*	−5.520	−0.588
30–60°	3.3 ± 0.7	2.7 ± 0.6	0.001*	−3.384	−0.361
60–90°	2.5 ± 0.7	2.1 ± 0.4	0.027*	−2.206	−0.235
90–120°	2.1 ± 0.6	1.9 ± 0.7	0.043*	−2.019	−0.215
>120°	1.7 ± 0.8	1.8 ± 0.7	0.579	−0.555	−0.059
**Non-dominant–Static**					
0–30°	8.3 ± 4.3	8.1 ± 3.5	0.870	−0.163	−0.017
30–60°	10.6 ± 6.1	10.6 ± 4.8	0.735	−0.338	−0.036
60–90°	7.5 ± 7.4	8.6 ± 6.9	0.138	−1.482	−0.158
90–120°	5.4 ± 5.2	5.4 ± 5.2	0.898	−0.128	−0.014
>120°	2.7 ± 2.8	4.1 ± 6.5	0.359	−0.918	−0.098
**Non-dominant–Dynamic**					
0–30°	1.9 ± 0.6	3.5 ± 1.2	<0.001*	−5.403	−0.576
30–60°	3.1 ± 0.8	2.8 ± 0.7	0.390	−2.066	−0.220
60–90°	2.6 ± 0.8	2.4 ± 0.6	0.294	−1.050	−0.112
90–120°	1.9 ± 0.4	1.9 ± 0.3	0.879	−0.152	−0.016
>120°	1.7 ± 0.7	1.7 ± 0.6	0.779	−0.280	−0.030

Bolded text and an asterisk next to the p-value indicate statistical significance at p < 0.05.

a Wilcoxon Signed Ranks Test.

* *Statically significant (p < 0.05)*.

There were significant effects of age (both cohorts) and PC-WUSPI scores for the MWC cohort on the average consecutive time static or dynamic in each humeral elevation range (Tables 5, 6). The MWC cohort had a moderate correlation between increased age and decreased average duration of consecutive dynamic periods in 0–30° of humeral elevation on the dominant side and 90–120° of humeral elevation on the non-dominant side (Table 5). These effects were not seen in the control cohort. There was a large association between increased PC-WUSPI scores and decreased consecutive dynamic periods in elevations above 120° on the dominant side (Table 6). However, there were no effects of presence of pain (binary variable) on the average consecutive duration static or dynamic in any humeral elevation range for either cohort.

**Table 5:** The correlation coefficient between age and the average consecutive duration of static and dynamic periods in each humeral elevation range for the MWC and control cohorts.

	**MWC**		**Control**	
	**Correlation coefficient^a^**	***p*-value**	**Correlation coefficient^a^**	***p*-value**
**Dominant–Static**				
0–30°	−0.178	0.249	−0.026	0.866
30–60°	0.091	0.559	−0.184	0.231
60–90°	0.130	0.402	−0.051	0.744
90–120°	0.094	0.542	−0.070	0.652
>120°	0.183	0.234	−0.056	0.720
**Dominant–Dynamic**				
0–30°	−0.394	0.008*	−0.136	0.378
30–60°	0.156	0.312	0.202	0.189
60–90°	0.127	0.412	−0.078	0.617
90–120°	−0.113	0.464	−0.315	0.038*
>120°	−0.117	0.450	−0.064	0.680
**Non-dominant–Static**				
0–30°	0.100	0.520	−0.076	0.622
30–60°	0.150	0.331	−0.019	0.904
60–90°	0.058	0.707	0.231	0.132
90–120°	0.155	0.316	−0.013	0.932
>120°	−0.090	0.562	0.018	0.907
**Non-dominant–Dynamic**				
0–30°	−0.215	0.161	−0.026	0.866
30–60°	0.200	0.193	−0.006	0.967
60–90°	−0.167	0.280	−0.129	0.404
90–120°	−0.331	0.028*	0.011	0.944
>120°	−0.031	0.839	−0.002	0.989

Bolded text and an asterisk next to the p-value indicate statistical significance at p < 0.05.

a Spearmans' Correlation.

* *Statistically significant (p < 0.05)*.

**Table 6:** The correlation coefficient between PC-WUSPI scores and the average consecutive duration of static and dynamic periods for the dominant and non-dominant arms in each humeral elevation range for the MWC cohort.

	**Dominant arm**	**Non-dominant arm**		
	**Correlation coefficient^a^**	***p*-value**	**Correlation Coefficient^a^**	***p*-value**
**Static**				
0–30°	0.038	0.830	−0.037	0.836
30–60°	0.061	0.730	0.323	0.063
60–90°	−0.079	0.656	0.132	0.457
90–120°	−0.119	0.503	0.180	0.308
>120°	−0.219	0.214	−0.076	0.671
**Dynamic**				
0–30°	0.099	0.579	0.006	0.974
30–60°	−0.061	0.733	0.150	0.397
60–90°	0.003	0.988	−0.042	0.813
90–120°	−0.109	0.541	0.123	0.489
>120°	−0.569**	**0.000**	0.052	0.771

Bolded text and an asterisk next to the p-value indicate statistical significance at p < 0.05.

a Spearmans' Correlation.

* *Statistically significant (p < 0.05)*.

## Discussion

The purpose of this study was to use wearable sensors in a remote data capture setting to understand the distribution of upper arm static and dynamic periods in five humeral elevation ranges for MWC users and matched able-bodied controls. We also aimed to understand the effects of covariates, such as age and pain (binary for both cohorts and continuous variable for the MWC cohort). The results of this study supported our hypotheses that different distribution of static and dynamic periods would be observed between cohorts across humeral elevation ranges, and that increasing age would be associated with decreased dynamic periods (although this was not seen at all humeral elevation ranges). We rejected our hypothesis that the presence of pain (binary variable) in both cohorts would be negatively associated with time dynamic across the humeral elevation ranges. However, the PC-WUSPI scores which quantify pain during activities related to wheelchair use (continuous variable) suggest that increased pain was associated with shorter durations of consecutive dynamic periods at high humeral elevation angles above 120° for the dominant arm.

Studies in workplace ergonomics have highlighted the risks of shoulder pain and disorders when working with hands above shoulder height (Lind et al., 2020). Specifically, increased exposure to upper-arm elevations over 60° and 90° have been indicated when evaluating risk for shoulder injury and pain (Ludewig and Cook, 2000; Svendsen et al., 2004; Dalbøge et al., 2016). This may highlight a specific risk for MWC users, who spent a higher percentage of daily time dynamic in humeral elevations over 60°. Dynamic activities in these humeral elevations specifically related to MWC use could include, but are not limited to, transfers and accessing standard height counters and shelves from a seated position (Finley et al., 2005; Kankipati et al., 2015). These tasks typically include loading and/or working with the arm away from the body, both of which are associated with increased risk (Koontz et al., 2012; Lind et al., 2020). In addition to more daily time in the 60–120° range, MWC users also exhibited longer consecutive periods of dynamic time in these humeral elevations. However, the difference of the daily time spent dynamic between cohorts is not large. For example, on the dominant side the MWC users spent an average of 12 min more dynamic per day in humeral elevations of 60–90° and three more minutes in humeral elevations of 90–120° than controls. It is not known yet whether an additional 12 or 3 min more in higher elevations would cumulate over days, months, and years to cause pain and pathology.

There were fewer differences in periods of static arm postures between cohorts than the dynamic time as described above. Both cohorts spent the highest percentage of their day static in 30–60° of humeral elevation; however MWC users spent significantly more time static in this humeral elevation range (42 min). Interpreting the clinical impact of this finding depends on whether the static time involves loading to the shoulder or rest to the musculature of the shoulder. If the shoulder is loaded then the increased static time observed among the MWC users may be one of the factors leading to increased pathology in this group. On the other hand, if this difference indicates a resting posture it could mean that either the dynamic time is a larger driver than the static time to shoulder pathology, or the static time required by the MWC group to limit pathology is larger than that for the able-bodied group. It is reasonable that MWC users' resting posture may fall within 30–60° due to wheelchair setup (arm rests and wheelchair handles can be used for support surfaces) or other postural effects (lower trunk soft tissue can protrude due to lack of innervation to abdominal musculature, thus arms rest on the body in a slightly abducted position). Further insight to whether the static time may be detrimental to MWC uses can be gleaned from considering the duration of static time events. No differences between groups were found in the average consecutive periods of static time in each humeral elevation range. For both cohorts, the humeral elevation ranges of <90° had longer static durations than those at higher elevations. The duration differences seen between humeral elevation ranges may provide insight into demands to the shoulder. A useful thought experiment is to consider activities of daily living and estimate how long the upper arm would be static and loaded simultaneously. There are likely few tasks which would be static and load the arm more than a few seconds; hence, we presume that the longer duration of static periods at lower elevations to be mostly unloaded.

As we hypothesized, increased age was associated with decreases in dynamic time for the MWC cohort, specifically in 30–60° of humeral elevation. Additionally, increased age was associated with less static time in 0–30° and increased static time in 30–60° in the MWC cohort. These results may indicate MWC users perform certain activities less frequently as they age. The associations between age and time static or dynamic in 30–60° of humeral elevation for the control cohort were either not significant or opposite the trends for MWC users. With only one matched pair over 60 years old in this cross-sectional study, our interpretations are limited with regards to aging with disability; however, our results do highlight decreased dynamic arm use in the humeral elevation range where wheelchair propulsion occurs.

Increased PC-WUSPI (shoulder pain) scores of MWC users were associated with decreased consecutive dynamic periods in elevations above 120° on the dominant side. Determining effects of pain on arm use is complicated because risk factors for pain development are largely unknown, shoulder pathology can exist with and without pain, and experts disagree about the source of symptoms (Lawrence et al., 2019). Our participants had an average PC-WUSPI score that is a fairly low level of pain; a previous study enrolled participants without pain as defined as WUSPI scores <12 (Mulroy et al., 2015). Mulroy et al. (2015) required an increase in the WUSPI score of 10 points to qualify as a participant who developed pain (Mulroy et al., 2015). Our sample consisted of participants whose function was not limited by their low levels of pain based on self-reports during their physical exam; therefore, interpretation of the association between PC-WUSPI scores and high elevation dynamic periods is unclear.

The results and interpretations presented here have limitations. Although this approach aims to capture a holistic view of a participant's kinematics, due to a limited sensor battery life (<12 h) and the inability for participants to wear the sensors when preforming wet daily activities, such as showering (the IMUs used in the study were not waterproof), some potentially risky periods of the day may be missed. Although we only aimed to measure kinematics, loads on the arm cannot be ignored when understanding potentially risky arm use since excessive load in any posture has been shown to be detrimental to the tendon (Lewis, 2010; Cardoso et al., 2019). This study did not measure loading or muscle activity during daily life. Current limitations in sensor technology and algorithms do not allow for the measurement/calculation of the plane of elevations that accompany the humeral elevation angles within the context of the field-based protocol we used. Yet, studies indicate that the plane of elevation affects the subacromial space for the tendon and the intramuscular pressure of the tendon (Järvholm et al., 1988; Palmerud et al., 2000; Lawrence et al., 2019), likely impacting risk to the tendons. Finally, a previous study found that up to 4 days of data collection are needed to reliably represent propulsion trends in a MWC user's daily life with a wearable accelerometer mounted on the wrist (Schneider et al., 2018). Additionally, a pilot study by our team showed that 4 days of data were needed to achieve good reliability to differentiate between arm use intensity levels (including static and dynamic) for MWC users (Goodwin et al., 2021). Only 1 or 2 days of data were collected for participants in this study due to participant availability and adherence to the protocol. We attempted to compensate for this by asking participants to wear the sensors on “typical days.” Lastly, a larger sample size would allow for analysis of additional important secondary predictors such as level of spinal cord injury and sex.

## Conclusion

This preliminary report expanded our understanding of how MWC users use their arms throughout a typical day using remote data capture practices. We sought to understand the daily percentage and average consecutive duration of periods MWC users and matched controls were static or dynamic in five humeral elevation ranges. Results indicated that on the dominant side MWC users spent a larger percentage of their day using their arm dynamically in the higher humeral elevation ranges of 60–90° and 90–120° than able-bodied controls which highlights the difference in performing activities of daily living from a seated position compared to able-bodied individuals who are able to stand. MWC users also had longer consecutive duration dynamic use periods in humeral elevations of 30–60°, 60–90°, and 90–120° than matched controls. For MWC users, age was significantly associated with increased static and decreased dynamic daily time in humeral elevations where manual wheelchair propulsion occurs (30–60°). Overall, these results point to potentially meaningful differences between MWC users and able-bodied individuals in how they use their arms to perform dynamic tasks between 30 and 120° of humeral elevation. Future studies will explore if these differences in dynamic use of the arm are associated with differences in pathology incidence and progression.

## Data Availability Statement

The raw data supporting the conclusions of this article will be made available by the authors, without undue reservation.

## Ethics Statement

The studies involving human participants were reviewed and approved by Mayo Clinic Institutional Review Board. Written informed consent was obtained from the individual for the publication of any potentially identifiable images or data included in this article.

## Author Contributions

BG was a part of acquisition, analysis and interpretation of data, drafting of the manuscript, statistical analysis, and technical support throughout the study. OJ was a part of acquisition, analysis and interpretation of data, critical review of the manuscript, and statistical analysis. SC was a part of data analysis, critical review of the manuscript, and technical support throughout the study. MV was a part of study conception, acquisition and interpretation of data, critical review of the manuscript, and obtaining funding. EF was a part of study conception, critical review of the manuscript, and technical support. MM was a part of study conception, interpretation of data, critical review of the manuscript, supervision, and obtaining funding. All authors contributed to the article and approved the submitted version.

## Conflict of Interest

The authors declare that the research was conducted in the absence of any commercial or financial relationships that could be construed as a potential conflict of interest.
