# Supplementary material for: Duration of Static and Dynamic Periods of the Upper Arm During Daily Life of Manual Wheelchair Users and Matched Able-Bodied Participants: A Preliminary Report
Source: Front Sports Act Living. 2021 Mar 26;3:603020. doi: 10.3389/fspor.2021.603020 (PMC8034231; doi:10.3389/fspor.2021.603020)

Supplemental Data

A) Participant demographics based on individuals who had one or two useable days of data.

	MWC cohort		Control cohort	
	**1-day of useable data**	**2-days of useable data**	**1-day of useable data**	**2-days of useable data**
**Age (years)**<br>Mean (SD)	44.1 (9.9)	42.3 (12.6)	43.8 (7.1)	42.7 (12.2)
**Sex**	5 females/6 males	5 females/28 males	1 female/3 males	9 females/31 males
**Self-reported weight (kg)**<br>Mean (SD)	75.5 (16.9)	78.8 (13.0)	91.9 (8.7)	81.5 (16.5)
**Self-reported height (cm)**<br>Mean (SD)	172.3 (7.2)	178.4 (8.1)	170.2 (7.0)	177.6 (10.1)
**Body mass index (kg/m^2)**<br>Mean (SD)	25.4 (5.4)	24.8 (3.9)	31.7 (2.1)	25.8 (4.2)
**Dominant arm**	8 right/3 left	28 right/5 left	4 right/0 left	35 right/5 left
**Injury Level**			-	-
Cervical (C6-C7)	2	6		
High/mid thoracic (T1-T8)	5	13		
Low thoracic/lumbar (T9-L1)	4	14		
**Time since injury (years)**<br>Mean (SD)	9.5 (10.4)	13.2 (11.7)	-	-
**WUSPI (dominant arm)**<br>Mean (SD)	13.9 (16.8)	15.2 (24.2)	-	-
**WUSPI (non-dominant arm)**<br>Mean (SD)	8.4 (13.2)	16.0 (22.7)	-	-

B) The percentage of the day spent static and dynamic in each humeral range for the MWC and control (Con) cohorts. * indicates $p < 0.05$ and ** indicates $p < 0.001$.

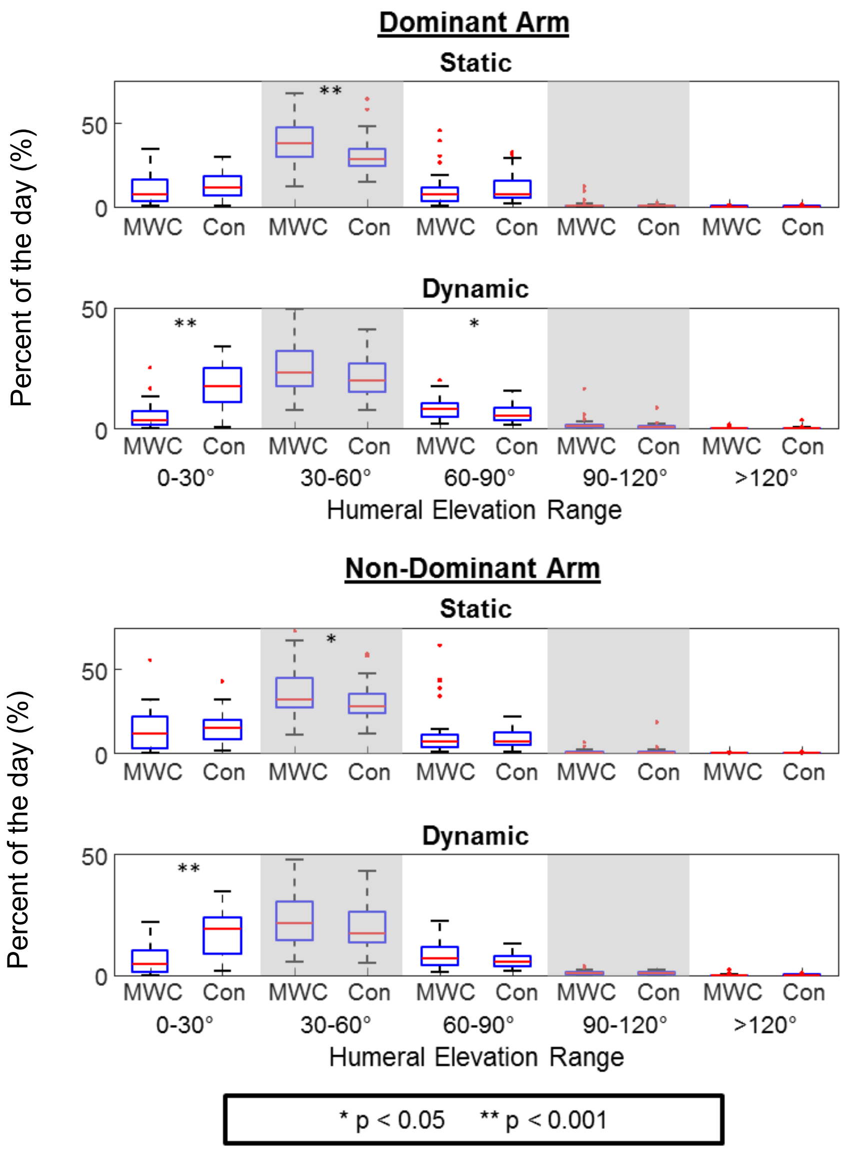

C) The association between age and the percentage of the day static and dynamic in humeral elevation bins on the dominant arm for MWC (red circles and red solid line) and control (blue squares and blue dashed line) cohorts. The Spearman's Correlation *rho* (ρ) denotes the strength of the association and the p-value shows the significance. These data expand on Figure 3.

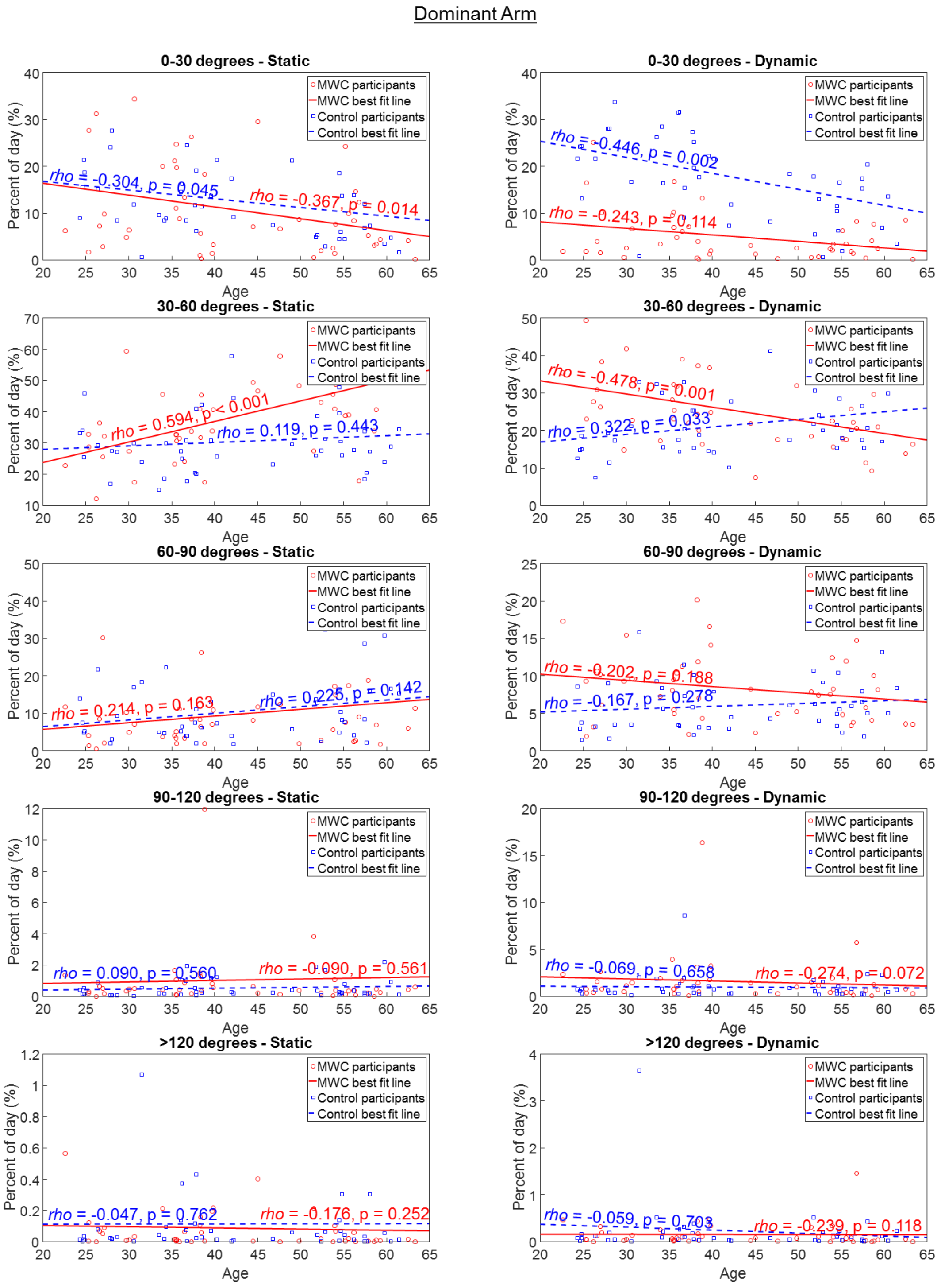

D) The association between age and the percentage of the day static and dynamic in humeral elevation bins on the non-dominant arm for MWC (red circles and red solid line) and control (blue squares and blue dashed line) cohorts. The Spearman's Correlation *rho* (ρ) denotes the strength of the association and the p-value shows the significance. These data expand on Figure 3.

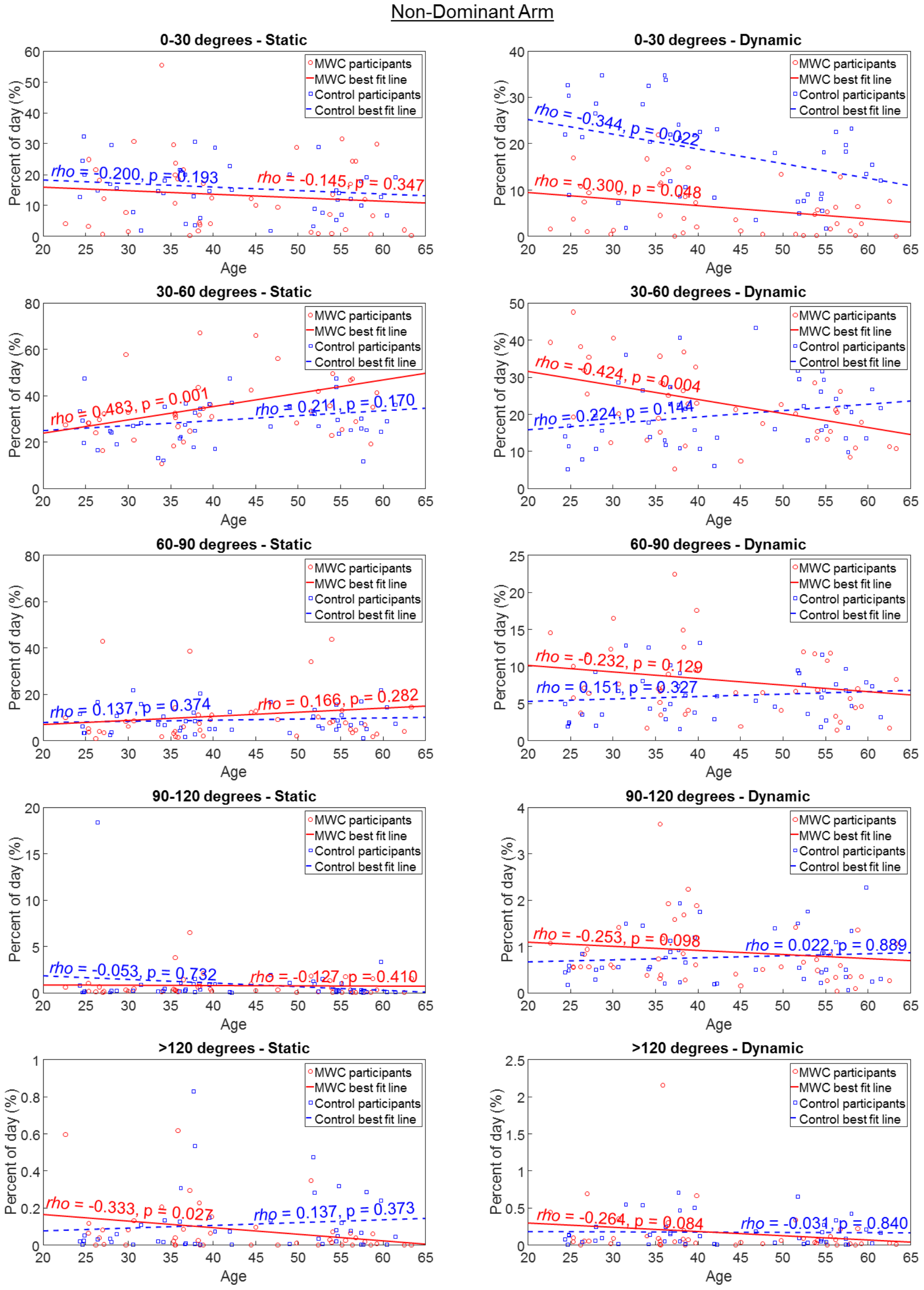

Supplement: Supplementary file 1 [file Data_Sheet_1.PDF]
